# Protocol of Kupffer phase whole liver scan for metastases: A single-center prospective study

**DOI:** 10.3389/fmed.2022.911807

**Published:** 2022-08-09

**Authors:** Qiong Wu, Yilun Liu, Di Sun, Yan Wang, Xiaoer Wei, Jing Li, Beibei Liu, Shuhao Wang, Yan Zhou, Haiyan Hu, Rui Zhang, Qiong Jiao, Yi Li, Tao Ying

**Affiliations:** ^1^Department of Ultrasound in Medicine, Shanghai Jiao Tong University Affiliated Sixth People's Hospital, Shanghai Institute of Ultrasound in Medicine, Shanghai, China; ^2^Institute of Diagnostic and Interventional Radiology, Shanghai Jiao Tong University Affiliated Sixth People's Hospital, Shanghai, China; ^3^Oncology Department, Shanghai Jiao Tong University Affiliated Sixth People's Hospital, Shanghai, China; ^4^Obstetrics and Gynecology Department, Shanghai Jiao Tong University Affiliated Sixth People's Hospital, Shanghai, China; ^5^Department of Pathology, Shanghai Jiao Tong University Affiliated Sixth People's Hospital, Shanghai, China

**Keywords:** hepatic metastases, conventional ultrasound, contrast-enhanced ultrasound, Sonazoid, Kupffer phase

## Abstract

**Introduction:**

As the presence of hepatic metastases is very important to cancer patients' clinical stage which would directly affect the selection and application of anti-cancer treatments. Although conventional ultrasound is commonly performed as a screening tool, most of the examinations have relatively poor sensitivity and specificity for detecting liver metastases. Contrast-enhanced ultrasound (CEUS) with Sonazoid has been reported to have the advantage of the diagnosis and therapeutic support of focal hepatic lesions and its specific Kupffer phase whole liver scan (KPWLS) is believed to be sensitive to detect liver metastases. And the purpose of this study is to determine the number, size, location and diagnosis of metastatic lesions, and to compare the results with conventional ultrasound and contrast-enhanced computed tomography (CECT), thus to clarify the application value, indications of Sonazoid-CEUS in screening liver metastasis.

**Methods and analysis:**

Kupffer phase whole liver scan for metastases (KPWLSM) is a self-control, blind map-reading, single-center, prospective superiority trial. Approved by the institutional review committee, the study period is planned to be from 1 January 2022 to 31 December 2025. Our study will include 330 patients with history of malignant tumors that cling to metastasize to liver. All patients will undergo the examinations of conventional ultrasound, Sonazoid-CEUS, and contrast-enhanced magnetic resonance imaging (CEMRI), and 65 of them should have additional CECT scans. The primary endpoint is the comparative analysis of the numbers of detected liver metastatic lesions among Sonazoid-CEUS, conventional ultrasound and CECT in screening liver metastases. Subjective conditions of patient after injection of Sonazoid will be followed up 3 and 30 days after KPWLSM, and any short-term and long-term adverse events are to be recorded with telephone interviews.

**Ethics and dissemination:**

This study has been granted by the Ethics Committee of Shanghai Jiao Tong University Affiliated Sixth People's Hospital (Approval No: 2021-197). When the KPWLSM is completed, we will publish it in an appropriate journal to promote further widespread use.

**Registration:**

Trial Registration Number and Date of Registration: Chinese Clinical Trial Registry, ChiCTR2100054385, December 16, 2021.

## Introduction

Liver is one of the most common sites for malignant tumor metastasis, and the presence, number, size, location and main adjacent relationship of hepatic metastases are crucial to the patient's clinical stage and the selection of overall treatment plan. Therefore, imaging examination of liver is very important for preoperative patients highly suspected with malignant tumor and in postoperative follow-up of patients with cancer that cling to metastasize to liver.

Contrast-enhanced US (CEUS) has been demonstrated as a suitable imaging technique because of higher spatial resolution and higher safety of microbubble contrast agents (1). Sonazoid (GE Healthcare, Oslo, Norway) is a highly specific mononuclear phagocytosis (MPS) US contrast agent which consists of perfuorobutane gas stabilized by a monomolecular membrane of hydrogenated egg phosphatidylserine (2). Sonazoid has been applied in various clinical practice including the diagnosis of focal liver lesions (3–5) and guidance of surgical or radiological interventions (6–8).

Traditional Sonovue ultrasound contrast agent has three vascular phases: arterial phase (10–40 s after intravenous administration), portal venous phase (60–90 s), and delay phase (3 min), and sonographers can observe the target lesion in these three phases in only 5 min. While Sonazoid contrast agents could be swallowed by macrophages of the liver (Kupffer cells), allowing for continuous imaging, which can last more than 1 h with high stability, then the sonographers could have sufficient time for a whole liver scan. In the Kupffer phase, malignant tumor, especially metastatic tumor will be present as a well-defined filling defect due to the lack of Kupffer cells which could be easily observed by the sonographer.

Although several studies have investigated the diagnostic performance of CEUS in liver metastasis, large heterogeneity is noted in their study results: such as sensitivity of 73–98.8%, specificity of 44.4–99.0%, different standards of reference (histology, magnetic resonance imaging (MRI), follow-up computed tomography (CT) and clinical follow-up), and inhomogeneous cohorts, etc. (9–12).

Besides, as Sonazoid became clinically available in April 2019 in China after prospective Phase 3 study conducted from May 2014 to April 2015 (13). A large-cohort and prospective study is still necessary to further explore the efficacy and indications of contrast Sonazoid-enhanced ultrasonography for the detection of hepatic metastases.

However, it is still unclear whether patients with malignant tumor truly benefit from KPWLS in terms of the detection of liver metastasis. We wonder whether it could be used as a screening tool for postoperative patients with malignant tumor to avoid unnecessary additional examinations such as contrast-enhanced computed tomography (CECT). And the purpose of this study is to determine the number, size and location of metastatic lesions, and to compare the results with conventional ultrasound and CECT, thus to clarify the application value, indications of Sonazoid CEUS in screening liver metastasis. We will also analyze how influential factors such as position, echogenity and other unknown factors restrict the use of Sonazoid CEUS and explore how to improve it.

## Methods and analysis

### Design

To make the study design more clear, we made a brief **PICOT** as follows:

### P (patient, population or problem)

Our study will include 330 patients with the history of malignant tumors prone to liver metastasis who visit the Shanghai Jiao Tong University Affiliated Sixth People's Hospital during this study period.

### I (intervention)

All patients will receive the examinations of Sonazoid-CEUS, conventional ultrasound and contrast-enhanced MRI (CEMRI), and 65 of them should have additional CECT scans.

### C (comparison or control)

Kupffer phase whole liver scan for metastases (KPWLSM) is a self-control, blind map-reading, single-center, prospective superiority trial. We set Sonazoid-CEUS, and CECT as the test group, and conventional ultrasound as the control group.

### O (outcome or objective)

**Figure 1:**
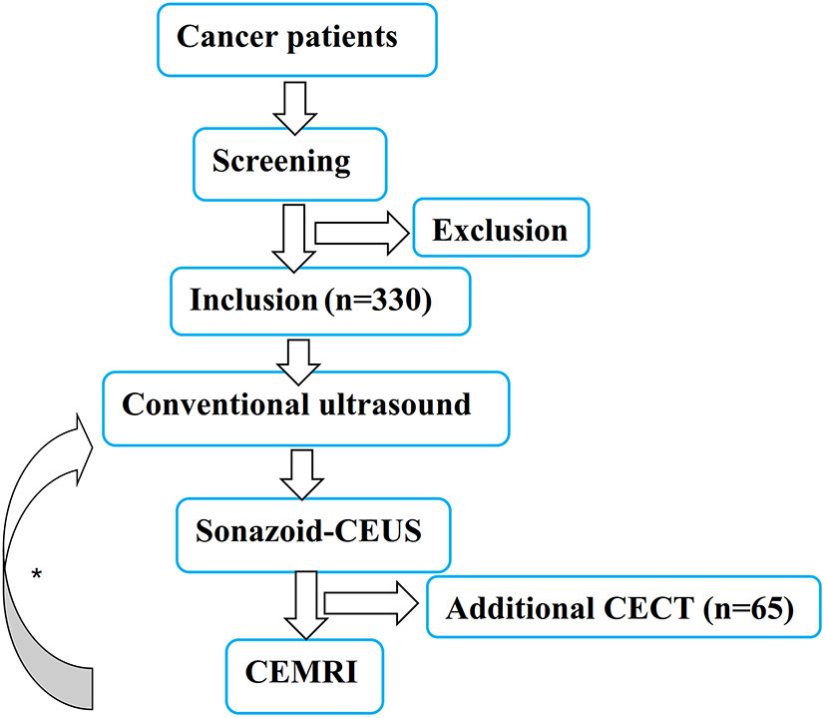
The study flow diagram. *Either radiological or ultrasound examination start first is OK.

The primary endpoint is the comparative analysis of the numbers of detected liver metastatic lesions among KPWLS in Sonazoid-CEUS, conventional ultrasound and CECT in screening liver metastases. The secondary endpoint is the comparative analysis of diagnostic accuracy between Sonazoid-CEUS and CECT in a single lesion. Subjective conditions of patient after injection of Sonazoid will be followed up 3 and 30 days after KPWLSM, and any short-term and long-term adverse events are to be recorded with telephone interviews. The study flow diagram is presented in Figure 1.

### T (time frame)

Approved by the institutional review committee, the study period is planned to be from 1 January 2022 to 31 December 2023.

### Study participants

#### Inclusion criteria

Inclusion criteria (must meet ①, ②, ③ and ④)

① The history of malignant tumors prone to liver metastasis (with or without specific liver metastasis, mainly non-epigastric malignancies);

② have done or plan to have a CEMRI examination within 2 months;

③ Age 18–75 y;

④ have done or plan to have a CECT examination within 2 months (optional, but at least 65 patients should finish it).

#### Exclusion criteria

① Patients with liver metastases after local treatment such as ablation or transcatheter arterial chemoembolization;

② Patients with a history of allergy to egg or any type of ultrasound contrast agent;

③ Pregnant or lactating women;

④ Reluctant to perform contrast-enhanced ultrasound examination;

⑤ Severe heart disease, hypertension, severe liver and kidney dysfunction;

⑥ Pulmonary dysfunction;

⑦ Other conditions that are not suitable for ultrasound examination or enhanced CT/MRI examination.

### Schedule of the study

After obtaining informed consent, the study group will check both of the inclusion and exclusion criteria, participants' background and pregnancy status. Four training cases and 330 formal cases were included consecutively, and all subjects will be subjected to conventional ultrasound, Sonazoid-CEUS, CEMRI examinations, 65 of which should also be subjected to CECT examinations.

### Case screening period

According to the inclusion and exclusion criteria, patients are included continuously, and the basic information is collected after signing the informed consent form. Child-bearing age women need to have negative urine human chorionic gonadotropin for excluding pregnancy before inclusion.

### After inclusion

This study is divided into radiological examination (CECT/CEMRI) and ultrasound examination (conventional ultrasound and KPWLSM). Both parts are completed independently in a blind state of reading images and filling out the case report forms (CRFs). Either radiological or ultrasound examination start first is all right, and the interval between the two examinations is required from 24 h to 60 days.

### Ultrasound examination

#### Conventional ultrasound

All patients in the group will undergo conventional ultrasound first, and once any lesion is detected, the number, size, location according to Couinaud's classification and sonographic features will be recorded. The ultrasound examination will be performed on PHILIPS EPIQ Elite (Philips Healthcare, Bothell, WA) with a 6-1MHz convex transducer.

#### Sonazoid-CEUS

CEUS acoustic power was at the default setting with a mechanical index of 0.18 and a dynamic range fixed at 45–65 db. Sonazoid was reconstituted in 2 mL sterile water for injection. The injection dose was 0.01 mL of encapsulated gas per kg of body weight. Sonazoid was injected as a bolus at a rate of 1 mL/s, followed by a 5 mL normal saline flush.

a Vascular phase images were obtained 0–5 min after Sonazoid injection. For the lesions found from conventional ultrasound, the vascular-phase characters of them would be recorded, Kupffer phase (10–15 min) will be observed, and the relevant characteristics will also be recorded on the CRF.
b If more than one nodule is found in pre-contrast conventional ultrasound, the largest one will be selected as target lesion, and more nodules should be observed at the same time if it is convenient in the same sonographic view. KPWLS were obtained at least 10 min after Sonazoid injection. If finding any additional defect in KPWLS, Sonazoid-CEUS would be repeated to observe the vascular character (for each patient, at most twice in one exam).
c The whole process of ultrasound examination will be completed by two senior sonographers with at least 5 years of US experience and 3 years of CEUS experience. Two senior sonographers will assess the enhancement patterns according to WFUMB-EFSUMB guideline (14).

#### Radiological examination

Patients may have CECT/CEMRI tests before or after ultrasound, and the interval should be between 24 h and 60 days. After completion of the examination, the images will immediately acquire blind desensitization treatment to ensure blinded reading. Then two senior radiologists will evaluate the images blinded to the other imaging.

#### CT

Liver CT will be performed on 320-detector scanners. Precontrast CT images will be obtained first, and then image acquisition in the arterial, portal venous, and delayed phases will begin 40, 70, and 180 s after intravenous administration of an iodinated contrast agent (iopamidol; Iopamiron 350, Nihon Schering, Osaka, Japan) at a dose of 100 mL and an infusion rate of 3 mL/s (15).

#### MRI

Liver MRI will be performed on 1.5 T or 3.0 T MRI scanners using a contrast agent (Gd -DTPA, Magnevist®, Bayer Health Care Pharmaceuticals, Berlin, Germany). All patients will accept standard MRI sequences, and the injection dose was 0.1 mmol Gd/kg body weight. Image acquisition in the arterial, portal venous phases, and late-dynamic phases will begin 10, 45, 120 and 180 s after intravenous administration of contrast agent at an infusion rate of 2 mL/s followed by a 30 mL saline flush.

#### Image assessment

Images of the lesions are to be saved as image files and the lesions should be marked with arrows. The readers will be asked to evaluate the whole liver images and pick up all lesions without any clinical information. When the consensus is reached after discussion, they will fill in the CRFs. The study coordinators will examine the correlation between these image files with their pathological and multi-imaging findings including follow-up imaging, and also collect all images and CRFs for specialized storage in the database.

#### Reference diagnosis

For patients undergoing hepatic surgery or biopsy, the final diagnosis for the target liver lesion in each patient will be determined based on the pathological results.

Not all cases have histological results, so a comprehensive clinical assessment including CEMRI, clinical or laboratory indicators is needed to determine whether the lesions are metastases. And the duration of follow-up for the controversial lesions is assigned as 1 year.

#### Primary endpoint

The primary study endpoint is the comparative analysis of the number of detected liver metastatic lesions among Sonazoid-CEUS, conventional ultrasound and CECT in screening liver metastases. If the results show that Sonazoid-CEUS could detect more lesion than conventional ultrasound and CECT, the study will be considered to be successful because it means the data collected in the present study confirmed the diagnostic improvement provided by Sonazoid-CEUS over conventional ultrasound and CECT, Sonazoid-CEUS will be confirmed as an excellent screening tool without radiation exposure.

#### Secondary endpoint

The secondary endpoint is the comparative analysis of diagnostic accuracy between Sonazoid-CEUS and CECT in a single lesion. When the results show that Sonazoid-CEUS provide better diagnostic efficacy than CECT, the study will be considered to be successful because it means the data collected in the present Sonazoid-CEUS could be a potential diagnostic tool instead of CECT despite the good diagnostic performances achieved by the latter.

#### Tertiary endpoint

The tertiary endpoint includes a detection rate of any adverse event related to Sonazoid-CEUS. The adverse event rate is defined as the proportion of patients having an adverse event following injection of Sonazoid out of the patients enrolled in the study. Short-term and long-term adverse events are to be recorded according to the Common Terminology Criteria for Adverse Events (CTCAE version 4.0) (16) with telephone interviews 3 and 30 days after KPWLSM, the patient with adverse events will be recalled to the hospital for further data collection. The study group will discuss each adverse event based on patient's medical history as to see if the adverse event is similar to that of other ultrasound contrast agents like SonoVue.

#### Stopping rule

The patient offers to withdraw.

The subject requested the withdrawal of informed consent.

From a medical point of view, the researchers considered it necessary for the subjects to discontinue the study, etc.

#### Sample size

As sample size calculation is closely related to research design, and this study consists of several parts which involves the comparison of superiority and diagnostic accuracy, so the calculation of multiple sample sizes is carried out, followed by the larger size as the final calculation results.

For the comparison of superiority and diagnostic accuracy between conventional ultrasound and Sonazoid-CEUS, we assumed a 25–50% prevalence of liver metastatic lesions in our target population (17), while 62.5–87.7% of sensitivity, 40–60% of specificity in conventional ultrasound (12), and 73–95% of sensitivity, 65–75% of specificity in Sonazoid-CEUS according to previous literature studies (18, 19). Also, a 10% dropout rate is included, and the required number of subjects is calculated to be 330.

For the comparison of superiority and diagnostic accuracy between CECT and Sonazoid-CEUS, we also assumed a 25–50% prevalence of liver metastatic lesions in our target population, while 90% of sensitivity in CECT (19), and 73–95% of in Sonazoid-CEUS according to previous literature studies. Since no AUC has been reported, this study attempted to use AUC of 0.75, 0.85, 0.95 for sample size calculation, and a 10% dropout rate is included. The required number of subjects for this part is calculated to be 65.

The sample size was calculated on the non-parametric module of PASS software using paired non-parametric test. With all these assumptions, a minimum of 330 patients are needed to obtain 80% or 90% statistical power for non-parametric test with an α equal to 0.05, of which at least 65 cases need to have received CECT.

#### Data management

For timely desensitization of personal information, once a patient has enrolled in research, he/she will get the number immediately which will become the unique identification without the appearance of their real name again during the whole clinical projects. Trained researcher will document in writing and submit the CRF to the expert committee for data checks in the accuracy and completeness. We will record detailed patient information, primary site status, treatment process, and response to anti-angiogenic therapy and immunotherapy. Relevant follow-up data will be collected with telephone interviews 3 and 30 days after KPWLSM. Using an electronic CRF, the collected data will be entered into the database for specialized storage. After all participants finishing the project, the dataset will be reviewed and analyzed accordingly.

### Data analysis

A commercially available software package (SAS version 9.2, SAS Analytics, Marlow, UK) will be used for all the statistical analysis, and the significance level was 0.05 for two-sided tests. Descriptive data will be mainly used in the study. Efficacy analyses of sensitivity, specificity, positive predictive value, negative predictive value and accuracy will be performed using the efficacy population including subjects who received Sonazoid-CEUS, conventional ultrasound, CECT and CEMRI in screening liver metastases.

For the comparison of superiority among Sonazoid-CEUS, conventional ultrasound and CECT, intra-individual comparison will be made in terms of the detection rate of the number of detected liver metastatic lesions with pared-samples *T*-test. And the Chi-square test or Fisher exact test will be used to compare the diagnostic accuracy of the three imaging modalities in screening liver metastases.

## Discussion

As the presence or absence of hepatic metastases are very important to cancer patients' clinical stage which would directly decide the selection and application of anti-cancer treatments (20). At present, the imaging modalities for liver include conventional ultrasound, CT and MRI. When the metastatic lesion is small or deep seated, or in segment 7/8, or the acoustic impedance difference between the lesion and hepatic tissue is not significant, the diagnosis is easily missed in conventional ultrasound. While CT also has a similar problem, due to the limitations of scan thickness and layer thickness, coupled with the patient's breathing artifact. There is also a certain risk of missing diagnosis when the density of small nodules is not significantly different from surrounding tissues, and this missed diagnosis cannot be corrected by repeated reading. In addition, as a screening tool that needs to be repeated in a certain follow-up cycle, patients need to bear a large radiation load from CT, and some of them even have allergic reactions to iodine used in CECT. Despite excellent sensitivity and specificity for detection of metastatic lesion, MRI is also not suitable to be a routine diagnostic tool in liver as it is not readily accessible in basic hospitals with high demands in breathing coordination and relatively high costs of time and money.

However, since there is no other better screening method in the clinical practice, CT and MRI are still considered as the main imaging modalities with high diagnostic efficacy for the detection of liver metastases in cancer patients. Although conventional ultrasound is commonly performed as a screening tool, most of the transabdominal ultrasound examinations have relatively poor sensitivity and specificity for detecting liver metastases. CEUS with Sonazoid has been reported to have the advantage of the diagnosis and therapeutic support of focal hepatic lesions using the specific Kupffer phase (21, 22). In the liver, macrophages acquire specific characteristics becoming Kupffer cells and working to ensure protection and immunotolerance. Angiogenesis is another double-edged sword in health and disease and it is the biggest ally of macrophages allowing its dissemination. As colorectal cancer is one of the most common cause of liver secondary tumors, cytotoxic T lymphocyte antigen-4 (CTLA-4) is an inhibitory immune checkpoint that can be expressed in tumor-infiltrating lymphocytes and colorectal cancer (CRC) cells. This immune checkpoint can attenuate anti-tumoral immune responses and facilitate tumor growth and metastasis. Although capecitabine is an effective chemotherapeutic agent for treating CRC, its effect on the tumoral CTLA-4 expression remains unclear (23). Thus, we would also record detailed patient information including not only primary site status, treatment process, but also the response to anti-angiogenic therapy and immunotherapy.

In 2016, a comparative study between CECT and Sonazoid-enhanced ultrasonography diagnosis of hepatic metastases in 148 nodules found that there was no statistical diagnostic difference between the two, and CEUS detected 19 occult lesions unrecognized by the use of CECT and 12 lesions were confirmed malignant by histopathology (12). Although several similar studies have investigated the diagnostic accuracy of CEUS in hepatic metastases, most of them are based on retrospective analyses of clinical data and radiologic findings (9, 24). Therefore, the present study would further verify the application value of Sonazoid-enhanced ultrasonography in screening liver metastasis prospectively. If the results show Sonazoid-CEUS provide better diagnostic performance than conventional ultrasound and CECT through diagnostic efficacy comparison, that would mean Sonazoid-enhanced ultrasonography has potential benefit in screening liver metastasis without having to refer cancer patients for additional CT or MRI.

Besides, as Sonazoid became clinically available in April 2019 in China after Japan, South Korea and other countries, several related domestic studies have been reported about the application value of Sonazoid for focal liver lesion especially in hepatocellular carcinoma, but few studies focused on metastatic liver tumors (25–27). A small-sample study with 27 patients (99 metastatic liver lesions) found contrast-enhanced intraoperative ultrasonography of Sonazoid may play a similar or even better role than other radiological methods (intraoperative ultrasonography and MRI) in diagnosing liver metastasis, and 8 occult metastatic lesions were newly found in 7 of the 27 patients, thus to change relevant treatment strategy (28).

With larger patient population, the current study will provide fundamental data of the size and location of metastatic lesions to explore the indications, application range of Sonazoid-CEUS in screening liver metastasis. We will also analyze how influential factors such as position, echogenity and other unknown factors eliminate the use of Sonazoid CEUS and explore how to improve it.

Reference diagnosis of our study is inevitably heterogeneous. Histopathological results will be obtained in some cases which is the strongest evidence of suspected lesions. However, not all enrolled patients will accept surgical resection or biopsy, in part of the cases may be based on comprehensive clinical assessment including CEMRI, clinical or laboratory indicators. A preferred 1-year follow-up will be performed to confirm the reference diagnosis in the controversial lesion.

KPWLSM is the first domestic large-sample study to investigate the diagnostic efficacy of Sonazoid-enhanced ultrasonography in screening liver metastasis among cancer patients. KPWLSM will provide strong evidence regarding whether patients with a tentative diagnosis of liver metastasis could truly benefit from Sonazoid-enhanced ultrasonography in terms of the detection of early liver metastasis while avoiding additional unnecessary MRI or CT.

## Ethics statement

The studies involving human participants were reviewed and approved by Ethics Committee of Shanghai Jiao Tong University Affiliated Sixth People's Hospital. The patients/participants provided their written informed consent to participate in this study.

## Author contributions

TY and YLi contributed to the conception and design of the trial. YW, XW, JL, BL, and SW performed the image interpretation. YZ, HH, RZ, and QJ made the patient inclusion and data acquisition. QW, YLiu, TY, and DS made substantial contributions to the organization of this trial. QW, YLiu, YLi, and DS were also involved in drafting the manuscript or revising it critically for important intellectual content. All authors have given final approval of the version of the protocol.

## Funding

This study was supported by college project of Shanghai Jiao Tong University Affiliated Sixth People's Hospital (grant number Ynts202104).

## Conflict of interest

The authors declare that the research was conducted in the absence of any commercial or financial relationships that could be construed as a potential conflict of interest.

## Publisher's note

All claims expressed in this article are solely those of the authors and do not necessarily represent those of their affiliated organizations, or those of the publisher, the editors and the reviewers. Any product that may be evaluated in this article, or claim that may be made by its manufacturer, is not guaranteed or endorsed by the publisher.
